# CRISPR-Cas gene editing technology and its application prospect in medicinal plants

**DOI:** 10.1186/s13020-022-00584-w

**Published:** 2022-03-04

**Authors:** Miaoxian Guo, Hongyu Chen, Shuting Dong, Zheng Zhang, Hongmei Luo

**Affiliations:** Institute of Medicinal Plant Development, Chinese Academy of Medical Sciences & Peking Union Medical College, Beijing, China

**Keywords:** CRISPR-Cas, Gene editing, Reverse genetics, Synthetic biology, Genetic improvement, Medicinal plants

## Abstract

The clustered regularly interspaced short palindromic repeats (CRISPR)-Cas gene editing technology has opened a new era of genome interrogation and genome engineering because of its ease operation and high efficiency. An increasing number of plant species have been subjected to site-directed gene editing through this technology. However, the application of CRISPR-Cas technology to medicinal plants is still in the early stages. Here, we review the research history, structural characteristics, working mechanism and the latest derivatives of CRISPR-Cas technology, and discussed their application in medicinal plants for the first time. Furthermore, we creatively put forward the development direction of CRISPR technology applied to medicinal plant gene editing. The aim is to provide a reference for the application of this technology to genome functional studies, synthetic biology, genetic improvement, and germplasm innovation of medicinal plants. CRISPR-Cas is expected to revolutionize medicinal plant biotechnology in the near future.

## Introduction

The traditional gene editing technology randomly integrates a target gene into a receptor genome, thus producing results with poor predictability and problems, such as gene silencing and unexpected variations. The targeted gene editing technology can precisely modify the locus information of a genome, achieve targeted gene deletion, insertion or replacement [1], and reduce impact on the receptor genome background. Thus, it is preferred by most biologists. In 2013, the third-generation clustered regularly interspaced short palindromic repeats (CRISPR)-Cas gene editing system was introduced, which corrected certain defects in the first- and second-generation gene editing systems based on the synthetic endonucleases zinc finger endonuclease (ZFN) and transcription activator-like effector nuclease (TALEN), such as the transfection inefficiency, design complexity and limitations on multiplexed mutations [2]. The CRISPR-Cas technology relies on the complementary pairing of guide RNA sequences with target DNA sequences to identify target sites, requiring only 20 nucleotide sequences to be artificially designed to target specific genes [3–5]. Owing to its strong technical advantages, CRISPR-Cas system instantly became a major area of interest within the field of molecular biology and has been successfully applied to targeted gene editing in many model plants and crops. However, the application of this technology to medicinal plants has not been extensively explored because of their complex genetic backgrounds, inefficient genetic transformation system and regeneration system.

Medicinal plants have been used for thousands of years, and bioactive natural compounds from medicinal plants play an important role in protecting health via the pharmaceutical and food industries, but they also represent important value in perfume, agrochemical, cosmetic industries [6]. With the accumulation of studies on medicinal plants, more and more high-quality reference genome and efficient transformation systems of medicinal plants have been established, such as *Salvia miltiorrhiza* [7], *Dendrobium officinale* [8], *Cannabis sativa* [9] and *Opium poppy* [10]. Scientists are increasingly focusing on mining critical genes in metabolic pathways and finding novel synthetic methods for increasing the production of effective compounds [11]. The application of the CRISPR-Cas system to gene functional studies and metabolic networks regulation of medicinal plants is essential and meaningful, presenting a promising method for improving quality and breeding ideal germplasms in medicinal plants.

Here, we review CRISPR-based tools and briefly introduce their research histories, structural characteristics, working mechanisms, and derivative tools and discuss how they are being used in medicinal plant gene editing. Finally, we conclude the potential of CRISPR technology as a tool for medicinal plant gene editing. CRISPR provides unprecedented opportunities for functional genome studies, synthetic biology, genetic improvement, and germplasm innovation of medicinal plants.

## Historical studies of CRISPR-Cas

CRISPR-Cas system is derived from the adaptive immune system formed by bacteria and archaea during long-term evolution. In 1987, a Japanese group discovered a special DNA sequence in the noncoding region of the alkaline phosphatase gene of *Escherichia coli* [12]. The sequence is composed of multiple repetitive DNA fragments in tandem. In 2002, this DNA sequence was dubbed short regularly spaced repeats [13, 14] and the name would later be changed to clustered regularly interspaced palindromic repeats (CRISPR) [15]. In 2005, it was found that CRISPR spacer sequences are highly homologous to the DNA sequences of viruses or foreign plasmids, suggesting that CRISPR may have a function specifically against infection by a foreign genetic material [16, 17]. In 2007, Barrangou et al. found that artificially changing repeats in CRISPR can regulate the immune ability of *Streptococcus thermophilus* to specific phage [18]. Through experiments, the CRISPR-Cas system was found to specifically recognize and obtain exogenous gene fragments that form an “immune memory”. When bacteria are re-infected with the same phage, the CRISPR-Cas system destroys exogenous genes and enables the bacteria to acquire resistance to this phage. In 2012, Jinek et al. found that a single-guide RNA in the CRISPR-Cas system was able to target specific DNA fragments and proposed that this system can be used in gene editing [19]. In 2013, Cong et al. successfully used the CRISPR-Cas system in the targeted gene editing of animal genomes [20]. Since then, the third-generation gene editing technology CRISPR-Cas was introduced and has been widely used in various fields of molecular biology because of its technical advantages.

## Structure of CRISPR-Cas

The CRISPR-Cas system comprises Cas gene family proteins and CRISPR array consisted of repeats, spacers, and the leader sequence. The leader sequence is located upstream of the CRISPR array and is responsible for the initiation of CRISPR transcription. Repeats are short repetitive sequences that are 21–48 nucleotides in length that can form a hair loop, and the number of repeats varies according to species, generally ranging from a few to several hundreds. Spacers are approximately 26–72 nucleotides and located between two repeats [21]. The coding sequence of the Cas gene is usually located in the upstream region of the CRISPR array and can encode a highly conserved nucleic acid-related Cas protein [22], which has a nuclease, helicase, and nickase and other activities and can specifically cleave DNA sequences [23].

## Working mechanism of CRISPR-Cas

**Fig. 1:**
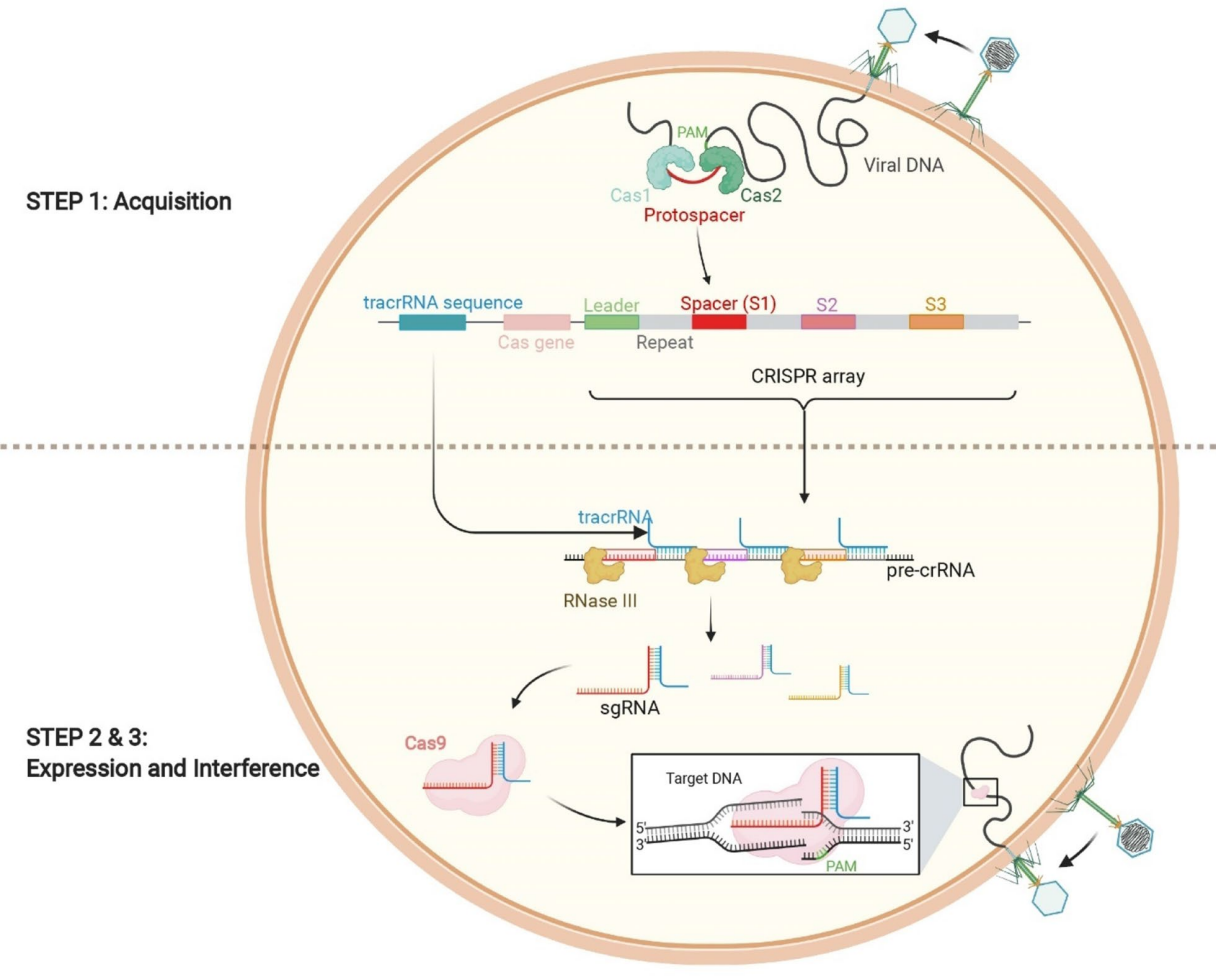
Schematic representation of CRISPR-Cas9 immunity. Step 1: Acquisition. Insertion of new spacers into the CRISPR locus. Step 2 and 3: Expression and interference. Transcription of CRISPR locus and processing of CRISPR-RNA, then recognition and degradation of foreign elements by the crRNA-Cas9 complex

The working mechanism of CRISPR-Cas system includes three steps: Acquisition, Expression and Interference (Fig. 1). The first stage is accomplished primarily by the complex of Cas1 and Cas2 proteins, which are shared by all known CRISPR-Cas systems, and sometimes involve additional Cas proteins. The protein complex recognizes the protospacer and protospacer adjacent motif (PAM) in foreign nucleic acids that are directionally captured and integrated as new CRISPR spacers into a CRISPR array separated by repeat sequences, thus creating an “immune memory” of invading genetic elements [17]. When the same exogenous gene is re-infested, the CRISPR locus is transcribed into a precursor CRISPR RNA transcript (pre-crRNA), which is then processed into a small mature crRNA, with the aid of Ribonuclease III (RNase III). The crRNA contains partial CRISPR spacer sequences joined to partial CRISPR repeat [24]. The CRISPR locus also encodes a trans-activating crRNA (tracrRNA) that has complementarity to the repeat regions of crRNA [25]. In addition to the CRISPR array, a single or multiple Cas nucleases are encoded by the CRISPR locus. For instance, in a type II CRISPR-Cas9 system, the most important feature is a large molecule protein Cas9, which participates in the maturation of crRNA and degrades invading exogenous nucleic acids. Fusing crRNA with tracrRNA produces the single-guide RNA (sgRNA) that complexes Cas9 [19]. Subsequently, the sgRNA binds to Cas9 to form an effector ribonucleoprotein complex responsible for the destruction of invading nucleic acids that are appropriately spaced from a required 5'-NGG-3' PAM sequence [26]. PAM is essential for recognition, cleavage, and distinction between self and non-self DNA [27–29]. Cas9 protein is characterized by two nuclease domains, RuvC and HNH, which perform cleavage functions; the HNH domain cleaves the complementary strand of the target DNA at a position three nucleotides upstream of the PAM sequence [19, 30], whereas the RuvC domain cleaves the other non-complementary strand at the same site, ultimately leading to exogenous DNA double strand breaks (DSBs) [19, 30].

**Fig. 2:**
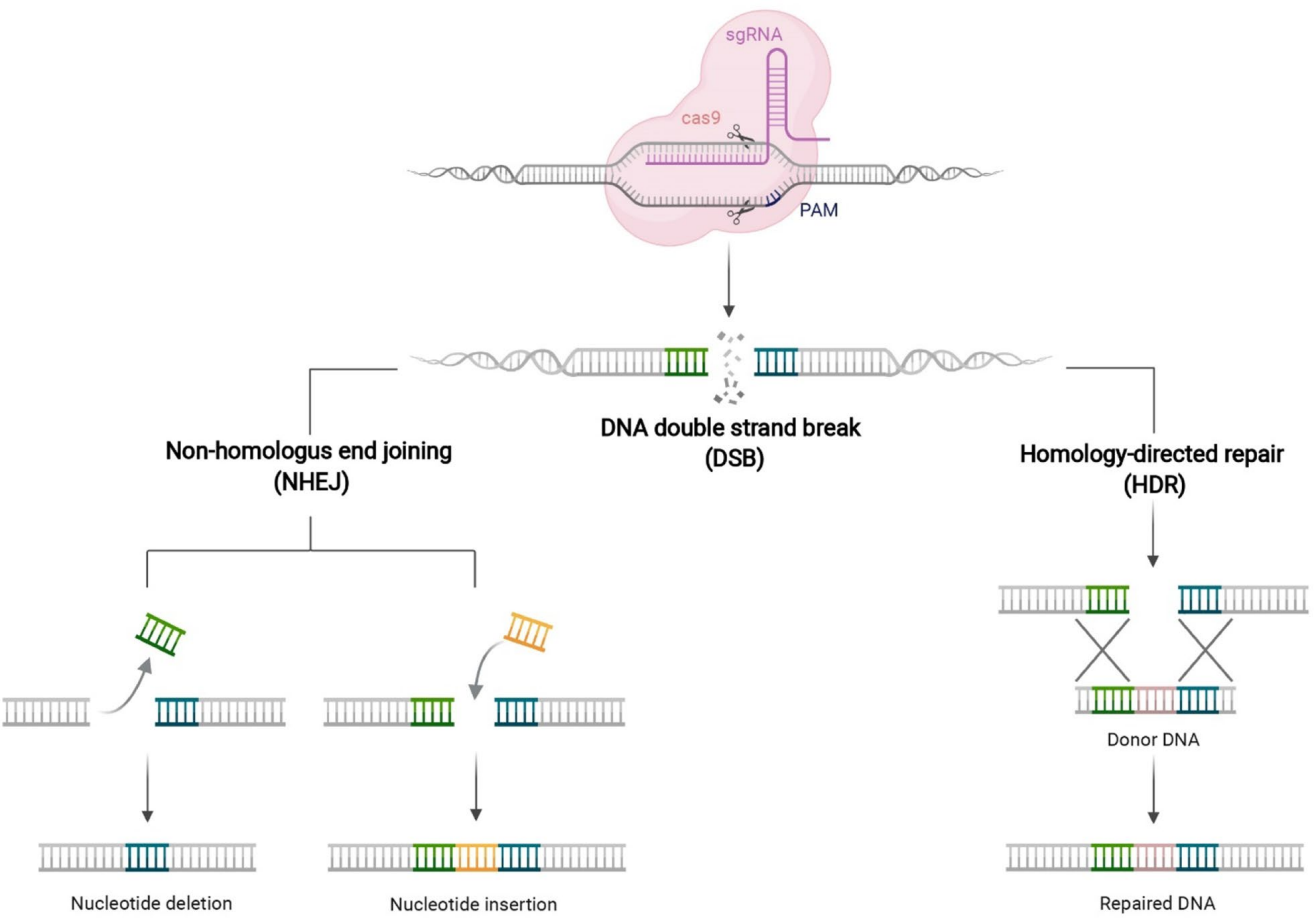
Genome editing with CRISPR-Cas9 systems can have multiple outcomes, depending on the DSB repair pathways: Nucleotide deletion and insertion are outcomes of the NHEJ repair pathway; Nucleotide modification precisely is outcomes of the HDR repair pathway using an available DNA donor template

Eukaryotic cells initiate DNA damage repair mechanisms, the most prominent being non-homologous end joining (NHEJ) and homology-directed repair (HDR), which can repair broken double-stranded gaps to achieve gene-targeted editing (Fig. 2). NHEJ is an error-prone mechanism that rejoins the two ends of a DSB with randomly frequent small nucleotide insertions or deletions, resulting in frameshift mutations and deletions, which in turn achieve targeted gene knockout. By contrast, HDR can achieve the precise editing of target genes, which allow the insertion or replacement of a specific nucleotide sequence in the presence of exogenous homologous donor templates. However, HDR-mediated gene targeting is challenging owing to the low spontaneous efficiency of HDR and the limitations of donor template delivery in cells [31].

## CRISPR-Cas novel systems and derivative tools

CRISPR systems are found in approximately 45% of bacteria and 85% of archaea and divided into two categories according to the configuration of their effector modules in the latest classification [32–34]. Class 1 effectors utilize multi-protein complexes, including type I, type III, and rarely, type IV, whereas Class 2 effectors rely on single-component effector proteins to disrupt target genes represented by Cas9, including types II, V, and VI [35–38].

**Fig. 3:**
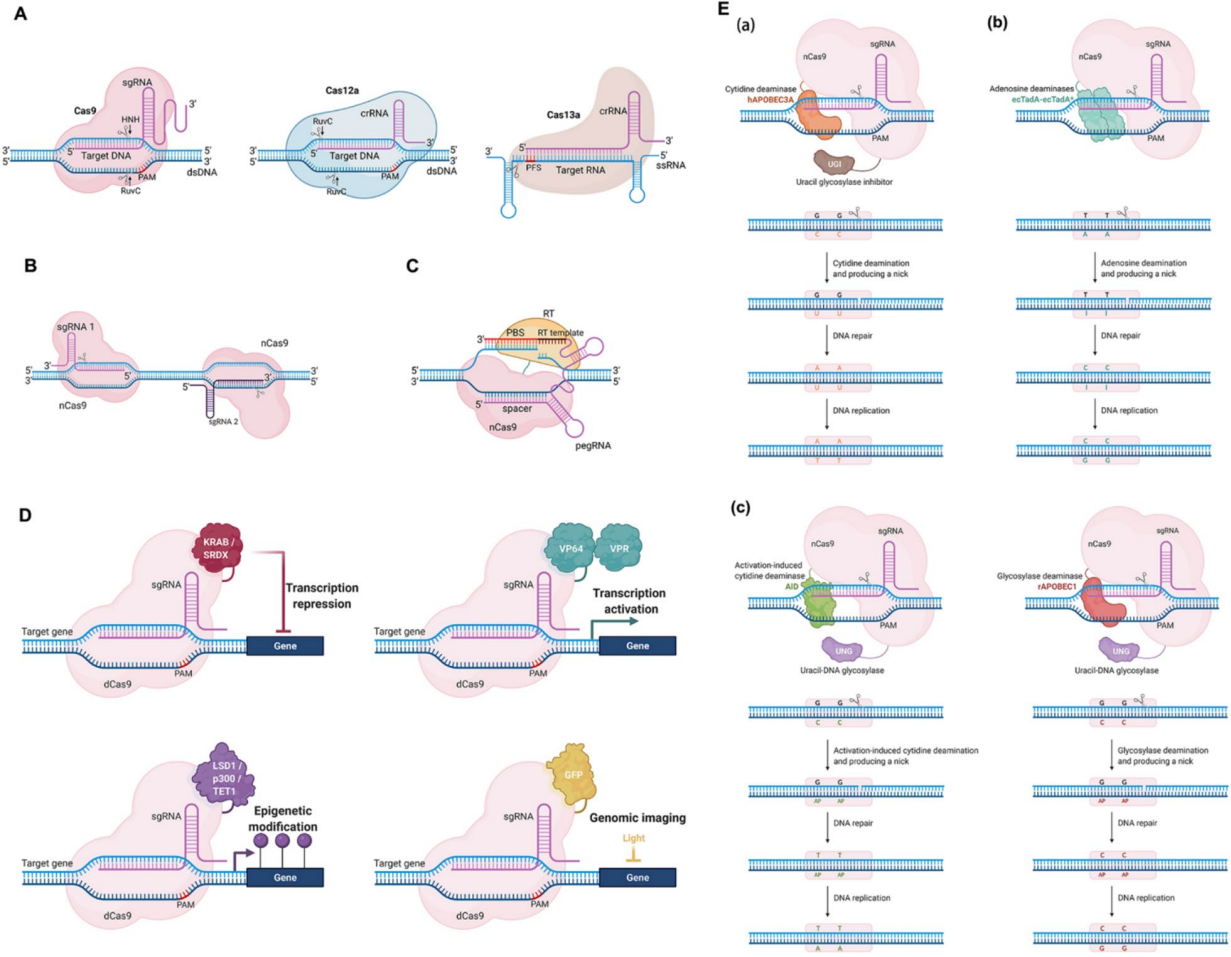
CRISPR-Cas systems for genome editing and other manipulations. **A** Schematic representation of representative three CRISPR-Cas systems: Cas9, Cas12a, and Cas13a. Their main features and action on the DNA/RNA are depicted. **B** Paired nickase system: Schematic representation of DBS by a pair of sgRNAs guiding Cas9 nickases. **C** Prime editor are generated through the fusion of nCas9 with an engineered reverse transcriptase (RT) and employment of a prime-editing guide RNA (pegRNA) that consists of the sgRNA containing a primer binding site (PBS) and the RT template sequence containing the desired edit. **D** Overview of various applications of dCas9 fusion-based genome manipulations. dCas9 fuses with other effector proteins, including transcriptional repressors (KRAB and SRDX) or activators (VP64 and VPR), epigenetic effectors (LSD1, p300, and ten-eleven translocation [TET1]), and fluorescent proteins (GFP) can be used for transcriptional modulation, epigenetic modification, and genomic imaging. **E** Mechanisms of single-base editing. **a** CBE-mediated C-to-T base-editing strategy. Cytidine deaminase is human APOBEC3A. **b** ABE-mediated A-to-G base-editing strategy. Deaminase is the fusion protein *Escherichia coli* TadA (transfer RNA adenosine deaminase). **c** GBE-mediated C-to-A and C-to-G base-editing strategy. The deaminases are activation-induced cytidine deaminase in *Escherichia coli* and rat APOBEC1 in mammalian cells

Diverse CRISPR systems are continuously identified in nature, and numerous novel CRISPR-Cas-mediated derivative technologies are created artificially. The toolbox of CRISPR base genetic editing is rapidly expanding (Fig. 3). Multiple CRISPR systems have been developed as efficient gene editing tools for DNA or RNA and applied to many fields.

### CRISPR-Cas9 variants

CRISPR-Cas9 belongs to type II in the second class of single-protein effector modules and is currently the most widely used and thoroughly studied genome editing tool. Multiple type II systems have been developed as efficient gene editing tools for DNA or RNA and applied to animals, plants, and microorganisms.

In 2013, *Streptococcus pyo*gene*s* Cas9 (SpCas9) was first used for genome editing in mammalian cells [20, 39]. It remains the most commonly used Cas9. The recognition of PAM 5′-NGG limits the availability of SpCas9 target sites for gene editing. For the expansion of the genome editing space of CRISPR and improvement of targeting specificity, CRISPR systems should be identified from new microbial species that may have different PAM requirements (Table 1). Another approach is to engineer Cas9 PAM specificities through structure-guided mutations and directed evolution (Table 2). These efforts have resulted in Cas9 variant proteins with small molecular weights and ability to recognize more PAM sequences. For instance, *Streptococcus thermophilus* Cas9 recognizes the PAM 5′-NNAGAAW (W represents A or T) [40]; *Neisseria meningitidis* Cas9 recognizes 5′-NNNNGATT [41, 42]; the expanded-PAM SpCas9 variant, SpRY, recognizes 5′-NRN and 5′-NYN (R represents A or C; Y represents C or T) [43], which can target almost all PAMs and may pave a path toward the development of editing technologies that are no longer constrained by inherent targeting limitations.

**Table 1:** Properties of CRRISPR-Cas9 orthologs

Cas9 orthologs	Native bacteria	PAM (5′ to 3′)	Size (amino acids)	Refs.
SpCas9	*Streptococcus pyogenes* Cas9	NGG	1368	[44, 45]
SaCas9	*Staphylococcus aureus* Cas9	NNGRRT	1053	[45, 46]
ScCas9	*Streptococcus canis* Cas9	NNG	1375	[47]
NmCas9	*Neisseria meningitidis* Cas9	NNNNGATT	1082	[43, 48]
CjCas9	*Campylobacter jejuni* Cas9	NNNNRYAC; NNNNACAC	984	[49, 50]
St1Cas9	*Streptococcus thermophilus* CRISPR1 Cas9	NNAGAAW	1121	[51]
St3Cas9	*Streptococcus thermophilus* CRISPR3 Cas9	NGGNG	1388	[51]
FnCas9	*Francisella novicida* Cas9	NGG	1629	[52, 53]
TdCas9	*Treponema denticola* Cas9	NAAAAN	1423	[54]
SmacCas9	*Streptococcus macacae* Cas9	NAA	1338	[55]
BlatCas9	*Brevibacillus laterosporus SSP360D4* Cas9	NNNNCND	1092	[56]
CasX	*Deltaproteobacteria* and *Planctomycetes phyla*	TTCN	980	[45, 57, 58]
CasY	*Katanobacteria*, *Vogelbacteria*, *Parcubacteria*, *Komeilibacteria* and *Kerfeldbacteria*	TA	1200	[47, 59]

**Table 2:** Properties of engineered CRRISPR-Cas9 variants

Engineered Cas9 variants	Included mutations	PAM (5′ to 3′)	Notes	Refs.
SpCas9 VRER	D1135V, G1218R, R1335E, T1337R of SpCas9 mutations	NGCG	Altered PAM variant; Bacterial-selection-based screening	[41, 60]
SpCas9 VQR	D1135V, R1335Q, T1337R of SpCas9 mutations	NGAN or NGNG	Altered PAM variant; Bacterial-selection-based screening	[41, 60]
SpCas9 EQR	D1135E, R1335Q, T1337R of SpCas9 mutations	NGAG	Altered PAM variant; Bacterial-selection-based screening	[61]
SpCas9-NG	R1335V, L1111R, D1135V, G1218R, E1219F, A1322R, T1337R of SpCas9 mutations	NG	Altered PAM variant	[62]
SpG	D1135L, S1136W, G1218K, E1219Q, R1335Q, T1337R of SpCas9 mutations	NGN	A near-PAMless variant	[43, 63]
SpRY	A61R, L1111R, N1317R, A1322R, R1333P introduced into SpG	NRN, NYN	A near-PAMless variant	[43, 63]
xCas9 3.7	E480K, E543D, E1219V, A262T, R324L, S409I, M694I of SpCas9 mutations	NG, GAA, GAT	Expanded PAM recognition range; Phage-assisted continuous evolution (PACE)	[25, 59]
SpCas9-HF1	N497A, R661A, Q695A, Q926A of SpCas9 mutations	NGG	Enhanced specificity	[64]
eSpCas9 (1.0)	K810A, K1003A, R1060A of SpCas9 mutations	NGG	Enhanced specificity; Structure-guided protein engineering	[65]
eSpCas9 (1.1)	K848A, K1003A, R1060A of SpCas9 mutations	NGG	Enhanced specificity; Structure-guided protein engineering	[65, 66]
evoCas9	M495V, Y515N, K526E, R661Q of SpCas9 mutations	NGG	Enhanced specificity; Yeast-based screening	[42]
HypaCas9	N692A, M694A, Q695A, H698A of SpCas9 mutations	NGG	Enhanced specificity	[67]
HiFi Cas9	single point mutation R691A of SpCas9	NGG	Enhanced specificity for ribonucleoprotein delivery	[68]
KKH SaCas9	E782K, N968K, R1015H of SaCas9 mutations	NNNRRT	Altered PAM variant	[69, 70]
SaCas-HF	R245A, N413A, N419A, R654A of SaCas9 mutations	NNGRRT	Enhanced specificity and genome-wide targeting accuracy	[69, 70]
efSaCas9	single point mutation N260D of SaCas9 variant Mut268	NNGRRT	Enhanced specificity; Human cells-based screening	[71, 72]
(HiFi-)Sc^++^	Thr1227Lys, Arg701Ala mutations and loop sequence from *S. anginosus* introduced into ScCas9	NNG	Enhanced specificity and activity	[73, 74]

### CRISPR/nCas9 and CRISPR/dCas9

The Cas9 protein has two domains, RuvC and HNH, which perform cleavage function. If a single base mutation (D10A or H840A) is introduced to one of the domains, Cas9 becomes nickase Cas9 (nCas9), which can only cleave a single strand in a target DNA sequence. If the two domains are mutated simultaneously, Cas9 becomes nuclease-deficient Cas9 (dCas9), which completely loses endonuclease activity. nCas9 and dCas9 have offered considerable advantage to the fields of transcriptional modulation, epigenetic modification, and genomic imaging.

nCas9 is often used in combination with two different sgRNAs for the simultaneous targeting of two single strands of a desired gene. This approach can significantly reduce the off-target effects of CRISPR-Cas9 systems and greatly improve the specificity of gene editing. nCas9 can be used for the replacement of large gene fragments and improvement of the probability of homologous recombination repair [75].

Although dCas9 loses nuclease activity, it still retains DNA-binding activity and can still target and bind to DNA sequences in a gRNA-programmable manner [76]. dCas9 regulates transcription by fusing transcriptional activators or repressors and modulating gene expression without introducing irreversible mutations into a genome [77, 78]. Approaches that use dCas9 for this purpose are commonly referred to as CRISPR activation (CRISPRa) and CRISPR interference (CRISPRi) [79]. CRISPRi [80] inhibits transcription through the aid of dCas-sgRNA complexes that sterically block RNA polymerase. CRISPRi has a significantly higher level of gene silencing than traditional RNAi technology [81]. CRISPRi occurs by inhibiting transcription, whereas RNAi degrades mRNAs in the cytoplasm. Notably, CRISPRi is sufficient for gene repression in bacteria, and auxiliary inhibitors are required to fuse it to dCas for chromatin-modifying transcriptionally repressive domains in eukaryotic cells, such as KRAB and SRDX domains [76, 77]. CRISPRa relies on the fusion of dCas9 to multiple repeats of transcriptional activation domains, such as VP64, VPR, p65AD, VP16, and VP160 [18, 79, 82], to enhance transcription at target sites. dCas9 can be applied to epigenetic modification and genomic imaging. dCas9 is combined with epigenetic effectors, such as histone demethylase LSD1, histone acetyltransferase p300, and TET proteins to modify epigenetic marks at their DNA or histone targets; this approach can alter the status of chromatin modification and regulate gene expression, cell differentiation, and other biological processes [83]. dCas9 is fused with fluorescent-labeled proteins, such as GFP, and can be used in visualizing DNA loci harboring repetitive sequences and labeling endogenous centromeres, pericentric regions, and telomeres with single or multiplex sgRNAs [84]. This approach generates a sgRNA site-specific imaging system and achieving visualize genomic loci in living cells in real time [37].

### Single-base editor and prime editor

Traditional CRISPR/Cas9 systems are all genetically edited by introducing DNA DSBs, which easily lead to excessive DNA damage or cells death [85]. In 2016, Komor et al. fused the cytosine deaminase with nCas9 or dCas9 for the first time to obtain a system that can efficiently achieve targeted nucleotide conversion from cytosine (C) to thymine (T) single base and named it cytosine single base editor, which can achieve the editing of the targeted gene without double-strand breaks and donor template [86]. Cellular DNA repair responses can antagonize this process and restore edited bases. Therefore, a uracil glycosylase inhibitor is used to prevent base excision repair and increase the efficiency of base editing [86–88]. In 2017, Gaydelli et al. successfully developed an adenine base editor that can accurately perform adenine (A) to guanine (G) nucleotide conversion with the aid of adenosine deaminases [89]. These two deaminases are later fused into a single engineered Cas9 protein, which can C-to-T and A-to-G base-editing activities [90–93]. A novel base editor has been added to the family: the glycosylase base editor, which can achieve nucleotide conversion from C to G [94, 95]. The advent of single base editors has offered the possibility of editing single specific bases that do not depend on HDR or donor DNA and do not involve the formation of DSBs, providing a highly efficient, simple, and universal technology for engineering nucleotide substitutions at target sites. The plant high-efficiency CBEs (PhieCBEs) produced by fusing the evolved cytidine deaminases with Cas9n-NG variants has been used in efficiently converting C to T in rice [96].

In 2019, Anzalone et al. successfully developed an ultra-precise novel gene editing tool, termed prime editor (PE), which fuses nCas9 with an engineered reverse transcriptase (RT) and uses a prime-editing guide RNA (pegRNA) [97]. pegRNA contains an sgRNA containing a primer binding site and an RT template sequence acting as a template for the creation of the desired edit in targeted DNA. The PE theoretically allows every possible base substitution and multiple base pair insertions, deletions, or combinations, effectively solving problems existing in single-base editor, which cannot modify all bases and have serious off-target effects, while greatly improving editing accuracy and expanding application scope of CRISPR.

### CRISPR/Cpf1 system

In 2015, Zetsche et al. found the type V subtype A CRISPR/Cpf1 system for the first time from *Acidaminococcus *sp. (AsCpf1) and *Lachnospiraceae bacterium* (LbCpf1) [98]. CRISPR/Cpf1 is mainly composed of two parts: Cpf1 protein (now known as Cas12a) and crRNA. Its working mechanism is similar to that of CPISPR/Cas9. The difference is that Cpf1-associated CRISPR arrays are processed into mature crRNAs without the requirement of an additional tracrRNA [25, 99]. The Cpf1-crRNA complex specifically targets exogenous DNA by recognizing a short T-rich PAM [100]. Subsequently, Cpf1 cleaves a 23-nucleotide complementary single strand and an 18-nucleotide non-complementary strand downstream of the PAM, which ultimately creates a five-nucleotide 5′ overhang [94, 101].

As a new member of the CRISPR system, CRISPR/Cpf1 expands editing sites beyond those of G-rich PAM preferred by Cas9. The generation of a staggered cut with an overhang provides an effective way for precisely introducing DNA into a genome through non-HDR mechanisms. crRNA (42 nucleotides) and Cpf1 (1307 aa) in CRISPR/Cpf1 have a lower number of nucleotides and smaller protein molecular weights than sgRNA (100 nucleotides) and SpCas9 (1368 aa) in CPISPR/Cas9 and thus more likely enter cells and simplify the design and delivery of genome editing tools. Given that Cpf1 is independent of other elements when it processes its own crRNA, it can be used in construct multiplexed genome editing [102]. More importantly, this system is highly sensitive to mismatches. One or two nucleotide mutations in a target sequence is sufficient to prevent cleavage [98, 99]. The advent of Cpf1 system brings new hope for breakthroughs in the CRISPR self-gene editing technology.

In 2016, Endo et al. successfully applied the CRISPR/Cpf1 system to plant genome editing for the first time [103]. However, owing to its narrow gene editing range, the applications of CPISPR/Cas9 are few. To address the limitations of the recognition of TTTV PAM alone by AsCpf1 and LbCpf1, variants have been engineered to recognize different PAMs. These variants include the AsCpf1 variant, which recognizes the PAMs 5′-TYCV and 5′-TATV (Y represents C or T; V represents A, C, or C) and the LbCpf1 variant, which recognizes the PAMs 5′-CCCC, 5′-TYCV, and 5′-TATG [104–106].

CRISPR-Cas12b (formerly C2c1) is a novel type V-B system derived from *Alicyclobacillus acidoterrestris*, *Alicyclobacillus acidiphilus*, *Bacillus thermoamylovorans*, and *Bacillus hisashii*. Similar to Cpf1, CRISPR-Cas12b prefers T-rich PAMs and produces DBS with 6–8 nucleotides sticky ends, and similar to Cas9, it requires the crRNA and trancrRNA. Cas12b has a small size, high temperature resistance, and high specificity, and thus has been used in engineering model plant genomes [107, 108].

### CRISPR-Cas13 system

One of the most recent discoveries in CRISPR-Cas is Cas13 (Cas13a, Cas13b, Cas13c, and Cas13d), which belongs to the Class 2 type VI group. Cas13 was first described in 2015 by Shmakov [35]. It was previously referred to as C2c2 (Cas13b termed C2c4, Cas13c termed C2c7) [109–111]. The simple structure of the CRISPR-Cas13 system comprises two components: the programmable single-effector RNA-guided RNase Cas13 and a crRNA, which just recognizes a target RNA by means of the protospacer-flanking site (PFS) analogous to the PAM sequence recognized by Cas9 [112, 113]. Furthermore, a novel type VI CRISPR-Cas13b from *Prevotella* sp*.* is more efficient than Cas13 and does not require any PFS [111]. What separates Cas13 from other predominant CRISPR-Cas systems, such as CRISPR-Cas9, is that it targets single-stranded RNA rather than double-stranded DNA. Cas13 proteins contain two higher eukaryotic and prokaryotic nucleotide-binding RNase domains (HEPN), which generate blunt ends in a target RNA after cutting [111–113]. RNA base editing using Cas13b was proposed by Zhang et al. in 2017. In this method, the adenine deaminase domain of ADAR2 that acts on RNA converting adenosine (A) to inosine (I) is fused with catalytically inactive Cas13b for RNA Editing for Programmable A to I Replacement (REPAIR) [111]. Then, cytidine (C)-to-uridine (U) RNA editor was developed, referred to as RNA Editing for Specific C-to-U Exchange (RESCUE) [114] by directly evolving ADAR2 into a cytidine deaminase and extending the RNA targeting toolkit. Furthermore, Cas13bt has been identified as the most ultrasmall family of Cas13b proteins, which have been used in REPAIR and RESCUE RNA editors and achieved the packaging of editors within a single adeno-associated virus [115]. CRISPR-Cas13 only edits full-length RNA transcripts and does not alter the DNA sequence, and thus it expands the power of CRISPR systems for gene engineering that requires short-term changes at the transcription level. Most importantly, it provides a robust, precise, and scalable RNA-targeting platform for RNA manipulation and has the potential to perform programmable RNA virus interference [116].

## Application of CRISPR-Cas technology to medicinal plants

The most used CRISPR-Cas system is the type II CRISPR-Cas9 system, and its applications in medicinal plants are mainly focused on few model plants with complete genome information and efficient genetic transformation systems (Table 3).

**Table 3:** Summary of the studies on CRISPR-Cas9-mediated medicinal plant gene editing

Species	Target gene	Gene description	Cas9/sgRNA promoter	Results	Mutation frequency	Refs.
*Salvia miltiorrhiza*	*SmCPS1*	*Committed diterpene synthase* gene in tanshinone biosynthetic pathway	CaMV 35S/AtU6-26	8 heterozygous and 3 homozygous hairy root mutants	11.5% and 30.8% for the homozygous and chimeric mutants	[119]
*Salvia miltiorrhiza*	*SmRAS*	*Rosmarinic acid synthase* gene in phenolic acid biosynthetic pathway	CaMV 35S/AtU6-26, OsU3	5 biallelic, 2 heterozygous and 1 homozygous hairy root mutants	50%	[120]
*Salvia miltiorrhiza*	*SmLACs*	*Laccase* genes in phenolic acid and lignin biosynthetic pathway	AtUBQ/AtU6	15 single-locus crispr lines and 14 dual-locus crispr lines	90.6%	[121]
*Salvia miltiorrhiza*	*SmbZIP2*	Basic leucine zipper transcription factor; negative regulator in phenolic acid biosynthetic pathway	CaMV 35S/AtU6-26	*SmbZIP2*-deficient hairy roots	12%	[122]
*Dendrobium officinale*	*C3H, C4H, 4CL, CCR,* and *IRX*	Involved in lignocellulose biosynthetic pathway	MtHP, CVMV, MMV, PCISV, CaMV 35S /OsU3	*DoLACs*-deficient hairy roots	16.7%, 20%, 33.3%, 33.3% and 6.7% for *C3H, C4H, 4CL, CCR* and *IRX*	[131]
*Dendrobium Chao Praya Smile*	*DOTFL1*	*Terminal flower 1* gene modulating flowering and inflorescence architecture	Ubi/OsU3, OsU6a	13 homozygous mutant plants	10.1%	[132]
*Cannabis sativa*	*CsPDS1*	*Phytoene desaturase* gene; Marker	CaMV 35S/AtU6	*CsPDS1*-deficient seedlings	2.5% and 51.6% for the homozygous and chimeric mutants	[139]
*Comfrey* (*Symphytum officinale* L. Boraginaceae)	*HSS*	*Homospermidine synthase* gene in PA biosynthetic pathway	–/AtU6-26	*HSS*-deficient hairy roots		[145]
*Opium poppy* (*Papaver somniferum* L.)	*4′OMT2*	*3′-hydroxyl-N-methylcoclaurine 4′-O-methyltransferase* gene in BIAs biosynthetic pathway	CaMV 35S /AtU6	*4′OMT2*-deficient mutant plants	85%	[146]
*Dioscorea zingiberensis*	*Dzfps*	*Farnesyl pyrophosphate synthase* gene	CaMV 35S /OsU3	9 *Dzfps*-deficient mutant plants	60%	[147]

### Application in *Salvia miltiorrhiza*

*Salvia miltiorrhiza* belongs to the Labiatae family, a traditional Chinese medicinal herb, and has been widely used in the treatment of cardiovascular and cerebrovascular diseases for thousands of year [117]. Its pharmacological activity is largely due to the presence of the lipid-soluble compounds known as tanshinones and water-soluble phenolic acids, including rosmarinic acid, salvianolic acid, and lithospermic acid [118]. Owing to its short life cycle, simple micropropagation methods, and efficient genetic transformation system, *S. miltiorrhiza* has been an ideal material for medicinal plant genetics and epigenetics research. After the dissection of its genome, it has become an emerging model plant for medicinal plant studies [7].

In 2017, Li et al. used CRISPR-Cas9 technology to precisely knock out *SmCPS1*, a committed diterpene synthase gene involved in main effective component tanshinone biosynthesis [119]. Three homozygous and eight chimeric transgenic hairy root mutants of *S. miltiorrhiza* were obtained through *Agrobacterium*-mediated transformation, and changes in the content and varieties of secondary metabolites between mutants and wild types were compared with the LC–MS technique. The predominant tanshinones included tanshinone I, tanshinone IIA, and cryptotanshinone, which were completely missing in homozygous mutants. This finding revealed that *SmCPS1* is a key gene of the tanshinone synthesis pathway, thus paving the way for subsequent studies on the secondary metabolic synthesis pathway of tanshinone and large-scale functional genome editing in *S. miltiorrhiza*. In 2018, Zhou et al. successfully transformed pure *S. miltiorrhiza* hairy root mutants through the targeted knock out of the rosmarinic acid (RA) synthase gene *SmRAS* in the water-soluble phenolic acid biosynthetic pathway by using the CRISPR-Cas9 system [120]. Five biallelic, two heterozygous, and one homozygous mutants were obtained from 16 independent transgenic hairy root lines. The levels of phenolic acids, including RA and salvianolic acid B (SAB), significantly decreased, whereas the levels of the RA precursor 3,4-dihydroxyphenyllactic acid clearly increased in the mutants. This result verified the function of *SmRAS*. In 2021, Zhou et al. knocked out more than 20 genes of the laccase family in *S. miltiorrhiza* simultaneously by using the CRISPR-Cas9 dual-locus editing technology [121]. The expression levels of the target laccase genes and phenolic acid biosynthesis key genes and the accumulation of RA, SAB and lignin decreased dramatically in the editing lines. Additionally, the growth and development of hairy roots were significantly retarded in the CRISPR lines. These results showed the function of *SmLACs*, which play key roles in development and lignin formation in the root of *S. miltiorrhiza* and are necessary for phenolic acid biosynthesis. This study provided a new strategy for functional studies and for targeting multiple genes and gene families. Shi et al. used overexpression (OE) and CRISPR-Cas9 technology to target *SmbZIP2*, a novel basic leucine zipper transcription factor isolated from *S. miltiorrhiza* [122]. Analyses on the transgenic lines revealed that phenolic acid content was elevated in the CRISPR-Cas9 lines but reduced in the OE lines. The research demonstrated that *SmbZIP2* is a negative regulator in phenolic acid biosynthesis, providing a novel biosynthesis strategy for phenolic acid production.

### Application in *Dendrobium officinale*

*Dendrobium officinale* belongs to the genus *Dendrobium* of Orchidaceae. It is one of the valuable medicinal herbs and has been applied to traditional medical herbal treatment for more than 2000 years. It possesses various pharmacological functions, such as hepatoprotective [123], anti-tumor [124], hypoglycemic [125], gastro-protective [126], and anti-inflammatory [127] functions. In 2020, *D. officinale* was listed as a dual-use plant with botanical medicine and food applications by the National Health Commission of China. However, the demand for *D. officinale* often exceeds the supply, and its price is extremely expensive because it has a low germination rate and slow growth and is overexploited [128]. Thus, it is very necessary and meaningful to use gene editing technology for research on functional genomics and breeding of new varieties possessing stable and fine inheritable characteristics in *D. officinale*. And the establishment of the high-quality whole-genome sequence of *D. officinale* published [8] and the transformation systems of the genus *Dendrobium* [129, 130] makes it possible to use CRISPR systems in *D. officinale* gene editing.

In 2017, Kui et al. successfully used CRISPR-Cas9 system in editing five targeted genes in the lignocellulose biosynthesis pathway*, coumarate 3-hydroxylase (C3H)*, *cinnamate 4-hydroxylase (C4H)*, *4-coumarate:coenzyme A liga*s*e (4CL)*, *cinnamoyl co-enzyme A reductase (CCR)*, and *irregular xylem5 (IRX)*, and measured the mutation rates of different target sites between 10 and 100% by using PCR amplification and sequencing techniques [131]. This study demonstrated that the CRISPR-Cas9-mediated genome editing system can be successfully applied to *D. officinale* genome editing, suggesting that the technology has great development potential as a tool for the genetic investigation and molecular breeding of *D. officinale*, even Orchidaceae. In 2021, Li et al. used a modified CRISPR-Cas9 technology to cleave and remove large genomic fragments in *Dendrobium* orchid *terminal flower 1* (*DOTFL1*) which can modulate flowering and inflorescence architecture, and successfully created its homozygous mutations in the T0 plants of *Dendrobium* Chao Praya Smile [132]. This study found the decreased expression of *DOTFL1* can accelerate flowering and pseudobulb formation and promote the early differentiation of the inflorescence meristem, and firstly established a link between the functions of genes and phenotypes in *D. officinale*.

### Application in *Cannabis sativa*

*Cannabis sativa* has a long history as a medicinal plant and is known for the pharmacological effects of cannabinoids, such as D9-tetrahydrocannabinol (THC) and cannabidiol (CBD), which have attracted renewed interest in recent years because of their therapeutic potential in the treatment of multiple human diseases, such as complex neurological diseases and cancer [133, 134]. Cannabinoids are investigated as potential therapeutic agents for COVID-19 [135]. *Cannabis*, as the most efficient natural source of secondary metabolite cannabinoids, has been widely used in therapeutic and industrial applications. Owing to increased demand for hemp-derived products, *Cannabis* has been one of the most economically valuable medicinal plants, and its growing market is expected to increase to 20.2 billion in 2020–2025 [136]. Thus, novel biotechnological tools are of great importance to the introduction of genetically modified hemp strains containing phytochemicals with improved quality and quantity. The CRISPR-Cas9 system has been highlighted for *Cannabis* genome editing because of its high target programmability and specificity. With the establishment of the high-quality reference genome of *Cannabis *de novo assembly [9] and efficient transformation systems [137, 138], CRISPR technology will play a more and more important role in the investigation of cannabinoid synthesis genes and genetic improvement in *Cannabis*.

In 2021, Zhang et al. employed the CRISPR-Cas9 technology to edit the phytoene desaturase gene (*CsPDS1*), a common marker gene that can be used in testing genetic manipulation tools, and finally generated four transgenic cannabis seedlings with an albino phenotype [139]. They also developed a stable transformation and regeneration method for transgenic cannabis plants. The stable *Agrobacterium*-mediated transformation system was constructed, and the stable integration of T-DNA in *cannabis* genome was validated. They used GRF3–GIF1 in the CRISPR vector, achieving a 1.7-fold increase in edited plant regeneration. This research can be applied to further functional genomic studies and demonstrated the potential of CRISPR gene editing in *C. sativa*.

### Application in comfrey

Comfrey (*Symphytum officinale* L. Boraginaceae) is a medicinal plant with anti-inflammatory, analgesic, and proliferative effects [140]. However, its pharmaceutical application is hampered by high contents of toxic pyrrolizidine alkaloid (PA) in the whole plant, which may result in hepatic toxicity in humans even at a low dose [141]. Despite the beneficial characteristics of comfrey’s valuable metabolites, its medicinal use is limited [142, 143]. Traditional sophisticated extraction or purification procedures are laborious and expensive [144], while the CRISPR-Cas9 gene editing technology might help reduce or ultimately shut down the biosynthesis pathway of toxic compounds from the source, providing raw materials for safe phytopharmaceuticals.

In 2020, Zakaria et al. used the CRISPR-Cas9 system to introduce detrimental mutations to the gene of homospermidine synthase (HSS), the first specific enzyme of the PA biosynthesis pathway [145]. HSS-deficient hairy roots (HRs) were successfully obtained, and the analysis showed that the levels of homospermidine and PA apparently deceased in the HRs. This research demonstrated the application potential of editing targeted gene and breeding low-toxic comfrey transgenic varieties with CRISPR-Cas genome-editing techniques.

## Application prospects of CRISPR in medicinal plants

**Fig. 4:**
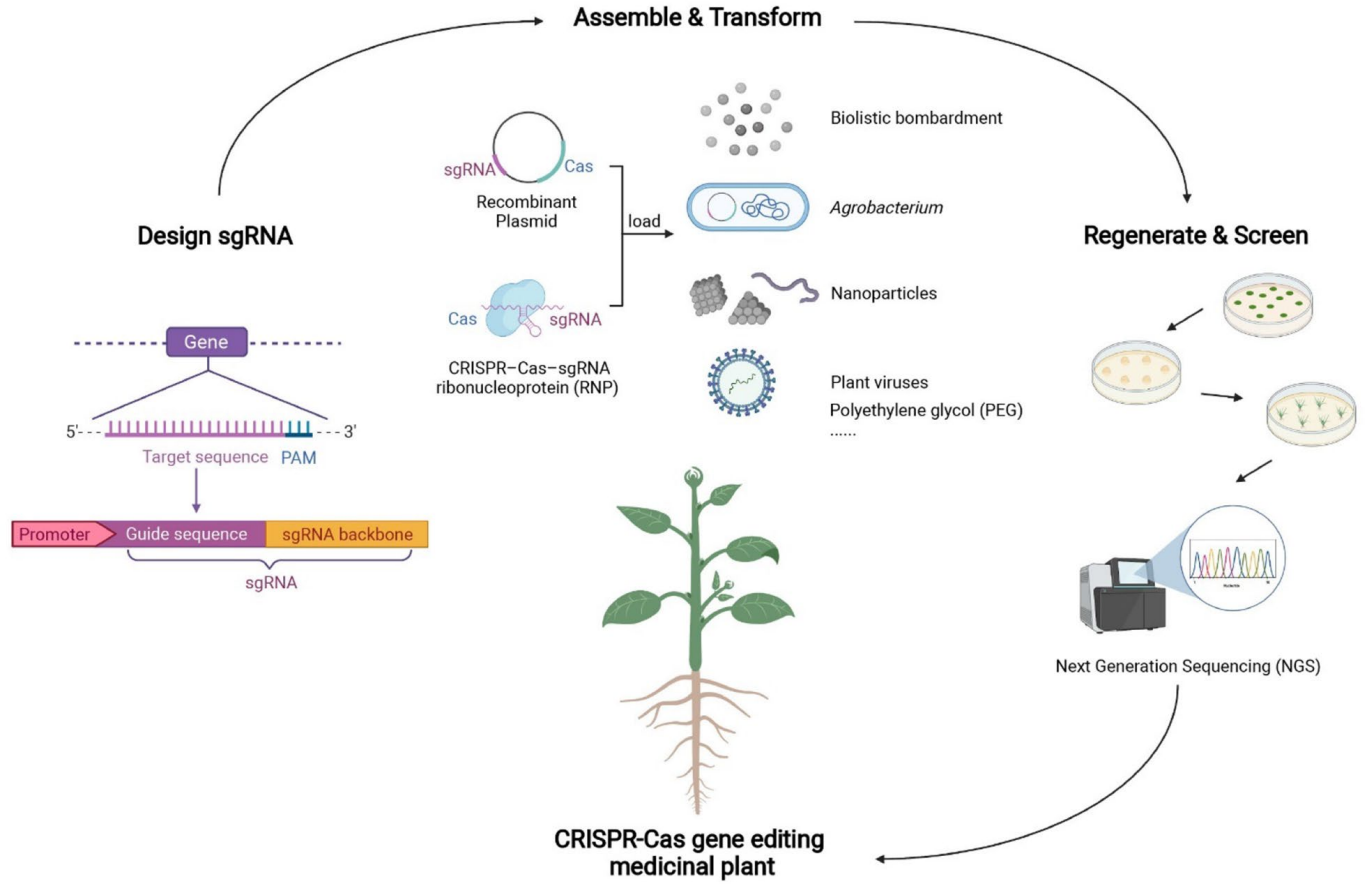
The pipeline of generating CRISPR/Cas-gene editing medicinal plant lines

CRISPR-Cas, as a third-generation gene editing technology, has the advantages of simple operation, short cycle, high efficiency, and wide scope of application, and it has been successfully applied to genome editing using medicinal plants, such as *S. miltiorrhiza*, *D. officinale*, *C. sativa* and so on (Fig. 4). The development of functional genomics and molecular biotechnology in medicinal plants has provided strong technical support to the application of the CRISPR-Cas gene editing technology. CRISPR is expected to promote scientific research and production application of medicinal plants in reverse genetics, synthetic biology, genetic improvement, and germplasm innovation.

### Reverse genetics of gene function

The genetic background of most medicinal plants and functional genes associated with the accumulation of important secondary metabolites are unclear at present, and CRISPR-Cas systems allows the rapid dissection of gene functions and gene–gene interactions from the perspective of reverse genetics. This technology can perform targeted knockout, insertion, replacement, and chromosomal recombination of genes at the DNA level, influencing the transcriptional expression of genes from the source. CRISPR is a powerful tool for verifying gene function in vivo.

The development of high-throughput sequencing technology has allowed the use of the CRISPR-Cas technology in dissecting gene function in key enzymes in the secondary metabolic pathways of medicinal plants. High-throughput sequencing technologies can detect changes in genes or their transcripts within an entire genome, transcriptome, or exome; screen potential key enzyme genes on a metabolic pathway; use the CRISPR-Cas technology to edit target genes; compare mutant plants obtained through genetic transformation with wild-type plants for secondary metabolite content detection or transcriptomics; and reverse verify their gene functions.

### Synthetic biology of effective components

The accumulation of effective components has always been a focus of research on medicinal plants. At present, the extraction of many important active Chinese medicine components still depends on the original plant which limited by deficient medicinal herb resources, laborious extraction and purification procedures, and variability due to weather and climate change. Synthetic biological products are effective ingredients that can be obtained by designing, regulating, and optimizing heterologous expression systems at the enzyme, metabolic pathway, and genomic levels. This approach addresses the above potential concerns. Additionally, the advent of CRISPR technology brings major advances in gene and pathway identification for effective constituent biosynthesis in medicinal plants, providing unprecedented opportunities for synthetic biology applications in active components production. Investigators leverage CRISPR-Cas technology for deeply mining key genes in the metabolic pathways of effective ingredients and precisely modifying biological engineering hosts, such as *Escherichia coli*, *Saccharomyces cerevisiae*, *Bacillus subtilis*, and tobacco cells, which have clear genetic background, rapid growth, and simple cultivation. The hosts are suitable for designing the metabolic pathways and biological elements of multiple modules by CRISPR technology that can be used in altering heterologous biosynthetic pathways, building cell factories, and generating precious active ingredients of medicinal plants. At present, these strategies have been successfully applied to research and production of artemisinin [148, 149], paclitaxel [150, 151], tanshinone [152, 153], and cannabinoids [154]. The production of bioactive natural products with synthetic biology strategies will reduce the overexploitation of medicinal plants to some extent and promote sustainable utilization of rare and valuable herbs.

### Genetic improvement and germplasm innovation

#### Improving quality

Improving quality is the primary target of gene editing using medicinal plants. The use of CRISPR-Cas gene editing technology in dissecting and regulating secondary metabolic biosynthetic pathways is essential to the improvement of the quality of medicinal plants. Two indexes are usually used in evaluating the quality of medicinal plants: physical indexes, mainly referring to appearance characteristics; chemical indexes, mainly referring to the types and contents of medicinal components and the contents of endogenous toxic substances and exogenous hazardous substances, such as chemical pesticide residues and toxic metal elements. Among them, the presence or absence of effective component in medicinal plants is the core element of the quality of medicinal plants and directly determines Traditional Chinese medicine clinical application effect. The CRISPR-Cas technology can improve the quality of medicinal plants from the following two aspects by regulating the metabolic synthesis network of active components. First, the CRISPR-Cas gene editing technology can activate the expression of transcription factors or key enzyme genes in the synthesis of effective components or downregulate them in the competitive pathway in order to increase the content of active components improving the intrinsic quality of medicinal plants. Second, the CRISPR-Cas technology can be used in knocking out genes related to the synthesis of toxic components or inhibiting their transcriptional expression for the reduction of the content of endogenous toxic and hazardous substances in medicinal plants, thus preventing the adverse reactions of medicinal plants.

In addition, the CRISPR-Cas gene editing technology can reduce ethical controversy about genetically modified products to a certain extent. Traditional transgenic technologies require the integration of foreign genes into receptor genomes to produce transgenic traits that can be stably inherited. However, the CRISPR-Cas technology requires no introduction of foreign gene fragments and only directs the modification of targeted genes. Furthermore, homozygous mutant lines without the Cas protein, sgRNA, and other exogenous genetic materials can be screened through self-crossing or hybridization, CRISPR-Cas-sgRNA ribonucleoprotein (RNP) element transient expression, and site-specific recombinant deletion technology. The new ‘the security of genetically engineered plants’ rule introduced in 2020 by the US Department of Agriculture to sustainably speed innovation in genetically engineered plant development. This guidance places genetically engineered plants under regulatory oversight only if they contain foreign DNA from agricultural pathogens. Thus, these CRISPR-Cas mutagenized plants are regulated on a ‘product-basis’ and do not fall under oversight by US regulatory agencies. Canada, Argentina, Brazil, Chile, Japan and Australia have similar regulatory frameworks [155]. A few CRISPR-edited plants have recently been introduced into this regulatory pipeline including corn, soybeans, mushrooms and camelina [156, 157]. New discoveries in CRISPR-Cas technology and continuous progress in delivery systems that do not need to insert any specific foreign DNA in host cells may bypass the strict biosafety legislative laws required for genetically modified products and thus has the production and application value of improving the quality of medicinal plants.

#### Improving yield

The yield of medicinal plants includes biological and economic yields. Biological yield is mainly the total dry matter yield formed through photosynthesis. Economic yield refers to the yield of medicinal parts. The use of the CRISPR gene editing technology in increasing the yield of medicinal plants can be conducted from two aspects. First, two reaction photosynthesis stages involved in the conversion of light energy into stable chemical energy of organic matter require the participation of certain enzymes, and corresponding enzyme gene is knocked out or overexpressed for the study of the function of genes, further regulation of gene expression, and improvement of photosynthetic products. Second, CRISPR can be used to regulate the expression of key functional genes controlling the growth of medicinal parts and increase of the yield of medicinal parts in medicinal plants.

#### Improving disease- and insect-resistance

Diseases and insect pests are the main factors affecting the safety and high-quality production of medicinal plants. The most economical and effective and safest way to control diseases and insect pests and promote the sustainable development of medicinal plants is to select and breed disease- and insect-resistant medicinal plants. The CRISPR-Cas technology provides a rapid way for generating germplasms with ideal resistance traits in medicinal plants. Researchers can follow relevant research methods to design CRISPR-Cas systems that can delete negative genetic elements or introduce gain-of-function mutations through precise genome editing in medicinal plants. They can also refer to known resistance gene-related crop studies and compare them with medicinal plant genomes to determine resistance homologous genes and directly edit these genes to create germplasms with beneficial disease- and insect-resistant traits. CRISPR can realize the simultaneous targeted editing of multiple genes controlling the key traits of medicinal plants and develop ideal medicinal plant varieties efficiently and accurately, greatly accelerating the speed of genetic improvement and germplasm innovation of medicinal plants.

#### Improving herbicide resistance

Weed damage is one of the main obstacle in the agricultural production of medicinal plants. Weeds compete with plants for growth space, water, and sunlight and spread pests and diseases directly or indirectly, thereby inhibiting the growth, reducing yields, and even seriously affecting the quality of medicinal plants. At present, the use of chemical herbicides is the primary method for controlling weeds because of their economic and good effects. However, most medicinal plants are extremely sensitive to herbicides, and spraying herbicides produce serious harmful effects resulting in seed germination rate reduction, leaf curling and yellowing, and even wilting and death. Medicinal plants can obtain resistance to certain herbicides through CRISPR gene modification and breed herbicide resistance medicinal plant varieties. *Acetolactate synthase*, which plays an important role in the synthesis of branched-chain amino acids. Directional mutation of its single amino acid, can reduce the sensitivity of plants to many chemical herbicides, such as sulfonylureas, imidazolinones, and pyrimidinylthiobenzoates [158]. *Acetyl coenzyme A carboxylase* is a crucial enzyme in lipid biosynthesis, and specific amino acid substitutions can cause aryloxyphenoxypropionate, cyclohexanedione, and phenylpyrazoline herbicides tolerance [159]. Glyphosate resistance can be achieved by the mutants of the *5-enolpyruvylshikimate-3-phosphate synthase* [160]. Other potential herbicide resistance genes, such as *protoporphyrinogen oxidase* [161], *tubulin alpha-2* [162], and *splicing factor 3B subunit 1* [163], confer resistance on butafenacil, trifluralin, and herboxidiene, respectively. Their roles can be further studied, and the result may lay a foundation for improving the herbicide resistance of medicinal plants. In addition, herbicide detoxification enzymes introduced into plants with the CRISPR technology reduce damage caused by herbicides to medicinal plants and cause herbicide resistance [164].

#### Accelerating the domestication of medicinal plants

More than 10,000 species of medicinal plants have been identified, but most medicinal plants are wild sources. Wild medicinal plants have some disadvantages, such as scattered distribution, serious habitat destruction, and poor yield stability, which restrict the sustainable supply and development of medicinal plants. Accelerating the domestication of medicinal plants is helpful in changing wild medicinal plants into domestic species, protecting endangered wild medicinal plant germplasm resources, and ensuring the sustainable utilization of traditional medicinal materials. In addition, this approach can facilitate the domestication of undesirable traits and further unify the cultivation and management of medicinal plants. The domestication cycle by traditional cultivation and breeding techniques is extremely long and involves changes in many loci. Few of these loci have key roles in driving the desired outcome. CRISPR-Cas, with its capacity for accurate genome manipulation, can undoubtedly accelerate the process of medicinal plant domestication. Pioneering studies of accelerated domestication have been conducted in crops. Li et al. modified genes related to shoot architecture, flower and fruit production, and ascorbic acid synthesis in tomato (*Solanum pimpinellifolium*) with the multiplex CRISPR-Cas9 technique [165]. Mutants were obtained with desirable traits on the premise of retaining parental disease resistance and salt tolerance, accelerating domestication of *S. pimpinellifolium*. Therefore, the advent of CRISPR technology facilitates the rapid domestication of medicinal plants and the development of wild medicinal plant varieties with excellent cultivation or agronomic traits, accelerating the modernization of Chinese medicinal materials.

## Conclusion and outlook

**Fig. 5:**
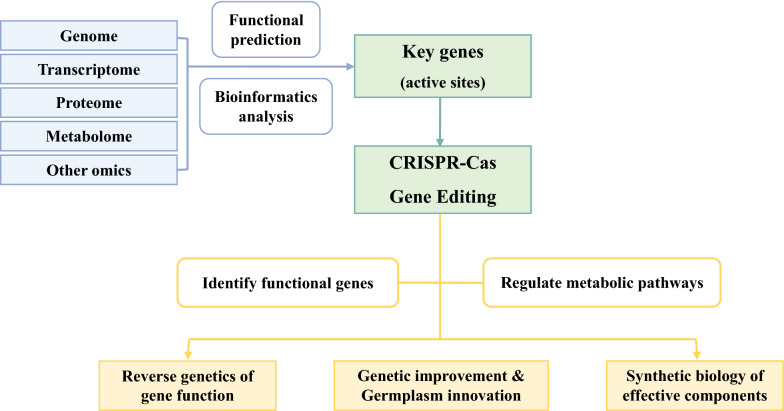
Diagram for CRISPR/Cas-gene editing strategies of medicinal plants combined with new omics technologies

The simplicity, versatility, robustness, and high target specificity of CRISPR-Cas and its derived editors make them powerful tools for precise medicinal plants gene editing through gene knockout, knock-in, replacement, point mutation, gene regulation, and modification at any gene locus, leading to tremendous advances in basic gene function research and genetic improvement in medicinal plants. With the successive completion of high-quality genome sequencing of medicinal plants, more and more important functional genes of medicinal plants can be used as target genes for gene editing. Taking *S. miltiorrhiza* as an example, the completion of the genome and transcriptome sequencing [7, 118, 166] and the in-depth analysis of the biosynthesis regulation pathway of the active components [122, 167–169] will provide abundant editing target sites for gene editing of *S. miltiorrhiza*. The development of omics technology will help provide more abundant target genes for the effective use of gene editing technology in medicinal plants, promote synthetic biology research on biosynthesis of bioactive compounds, genetic improvement of medicinal plant development and germplasm innovation (Fig. 5). Although CRISPR-Cas holds great promise in medicinal plants gene editing, the process still includes several bottlenecks. Given that precise knowledge of functional genomics is required for gene editing, additional studies are needed to obtain high-quality genomic information of medicinal plant species combined with the latest sequencing technology. Furthermore, delivering CRISPR-Cas reagents to cells and subsequently regenerating medicinal plants remain difficult. Hence, robust transform methods must be developed, particularly those that use carbon nanotubes [170, 171], DNA nanosturctures [172], cell-penetrating peptides [173] and plant viruses [174], which can efficiently diffuse into the medicinal plant cell wall without mechanical aid and without causing tissue damage. Finally, as producing an ideal cultivar requires the alteration of several quantitative traits and editing individual genes may not produce sufficient phenotypic change, efficient multiplexed genome editing methods for medicinal plants are needed.

Overall, owing to the rapid progress in CRISPR gene editing technologies, sequencing technologies, transformation and regeneration technologies, and many other related techniques, CRISPR-Cas will continue to revolutionize medicinal plant biotechnology in the near future.

## Data Availability

Not applicable.

## Abbreviations

CRISPR: Clustered regularly interspaced short palindromic repeats
PAM: Protospacer adjacent motif
pre-crRNA: Precursor CRISPR RNA transcript
tracrRNA: Trans-activating crRNA
sgRNA: Single-guide RNA
DSBs: Double strand breaks
NHEJ: Non-homologous end joining
HDR: Homology-directed repair
RT: Reverse transcriptase
pegRNA: Prime-editing guide RNA
PBS: Primer binding site
nCas9: Nickase Cas9
dCas9: Nuclease-deficient Cas9
CRISPRa: CRISPR activation
CRISPRi: CRISPR interference
PE: Prime editor
PFS: Protospacer-flanking site
HEPN: Higher eukaryotic and prokaryotic nucleotide-binding RNase domains
REPAIR: RNA Editing for Programmable A to I Replacement
RESCUE: RNA Editing for Specific C-to-U Exchange
RA: Rosmarinic acid
SAB: Salvianolic acid B
PA: Pyrrolizidine alkaloid
RNP: CRISPR-Cas-sgRNA ribonucleoprotein

## References

[1] Ceasar SA, Rajan V, Prykhozhij SV, Berman JN, Ignacimuthu S (2016). Insert, remove or replace: a highly advanced genome editing system using CRISPR/Cas9. Biochim Biophys Acta.

[2] Doudna JA, Charpentier E (2014). Genome editing. The new frontier of genome engineering with CRISPR-Cas9. Science..

[3] Lozano-Juste J, Cutler SR (2014). Plant genome engineering in full bloom. Trends Plant Sci.

[4] Liu L, Fan X-D (2014). CRISPR-Cas system: a powerful tool for genome engineering. Plant Mol Biol.

[5] Mahfouz MM, Piatek A, Stewart CN (2014). Genome engineering via TALENs and CRISPR/Cas9 systems: challenges and perspectives. Plant Biotechnol J.

[6] Hassan B (2012). Medicinal plants (importance and uses). Pharm Anal Acta..

[7] Xu H, Song J, Luo H, Zhang Y, Li Q, Zhu Y (2016). Analysis of the genome sequence of the medicinal plant *Salvia miltiorrhiza*. Mol Plant.

[8] Niu Z, Zhu F, Fan Y, Li C, Zhang B, Zhu S (2021). The chromosome-level reference genome assembly for *Dendrobium officinale* and its utility of functional genomics research and molecular breeding study. Acta Pharm Sin B.

[9] Gao S, Wang B, Xie S, Xu X, Zhang J, Pei L (2020). A high-quality reference genome of wild *Cannabis sativa*. Hortic Res.

[10] Guo L, Winzer T, Yang X, Li Y, Ning Z, He Z (2018). The *opium poppy* genome and morphinan production. Science.

[11] Kiss AK, Piwowarski JP (2018). Ellagitannins, gallotannins and their metabolites- the contribution to the anti-inflammatory effect of food products and medicinal plants. Curr Med Chem.

[12] Ishino Y, Shinagawa H, Makino K, Amemura M, Nakata A (1987). Nucleotide sequence of the iap gene, responsible for alkaline phosphatase isozyme conversion in *Escherichia coli*, and identification of the gene product. J Bacteriol Am Soc Microbiol.

[13] Mojica FJ, Díez-Villaseñor C, Soria E, Juez G (2000). Biological significance of a family of regularly spaced repeats in the genomes of archaea, bacteria and mitochondria. Mol Microbiol.

[14] Jansen R, van Embden JDA, Gaastra W, Schouls LM (2002). Identification of genes that are associated with DNA repeats in prokaryotes. Mol Microbiol.

[15] Mojica FJM, Garrett RA, Barrangou R, van der Oost J (2013). Discovery and seminal developments in the CRISPR Field. CRISPR-Cas systems: RNA-mediated adaptive immunity in bacteria and archaea.

[16] Mojica FJM, Díez-Villaseñor C, García-Martínez J, Soria E (2005). Intervening sequences of regularly spaced prokaryotic repeats derive from foreign genetic elements. J Mol Evol.

[17] Pourcel C, Salvignol G, Vergnaud G (2005). CRISPR elements in *Yersinia pestis* acquire new repeats by preferential uptake of bacteriophage DNA, and provide additional tools for evolutionary studies. Microbiology.

[18] Barrangou R, Fremaux C, Deveau H, Richards M, Boyaval P, Moineau S (2007). CRISPR provides acquired resistance against viruses in prokaryotes. Science.

[19] Jinek M, Chylinski K, Fonfara I, Hauer M, Doudna JA, Charpentier E (2012). A programmable dual-RNA-guided DNA endonuclease in adaptive bacterial immunity. Science.

[20] Cong L, Ran FA, Cox D, Lin S, Barretto R, Habib N (2013). Multiplex genome engineering using CRISPR/Cas systems. Science.

[21] Grissa I, Vergnaud G, Pourcel C (2007). The CRISPRdb database and tools to display CRISPRs and to generate dictionaries of spacers and repeats. BMC Bioinform.

[22] Richter H, Randau L, Plagens A (2013). Exploiting CRISPR/Cas: interference mechanisms and applications. Int J Mol Sci.

[23] Bland C, Ramsey TL, Sabree F, Lowe M, Brown K, Kyrpides NC (2007). CRISPR recognition tool (CRT): a tool for automatic detection of clustered regularly interspaced palindromic repeats. BMC Bioinformatics.

[24] Barrangou R (2015). Diversity of CRISPR-Cas immune systems and molecular machines. Genome Biol.

[25] Deltcheva E, Chylinski K, Sharma CM, Gonzales K, Chao Y, Pirzada ZA (2011). CRISPR RNA maturation by trans-encoded small RNA and host factor RNase III. Nature.

[26] Garneau JE, Dupuis M-È, Villion M, Romero DA, Barrangou R, Boyaval P (2010). The CRISPR/Cas bacterial immune system cleaves bacteriophage and plasmid DNA. Nature.

[27] Marraffini LA, Sontheimer EJ (2010). Self versus non-self discrimination during CRISPR RNA-directed immunity. Nature.

[28] Mojica FJM, Díez-Villaseñor C, García-Martínez J, Almendros C (2009). Short motif sequences determine the targets of the prokaryotic CRISPR defence system. Microbiology.

[29] Horvath P, Romero DA, Coûté-Monvoisin A-C, Richards M, Deveau H, Moineau S (2008). Diversity, activity, and evolution of CRISPR loci in *Streptococcus thermophilus*. J Bacteriol.

[30] Gasiunas G, Barrangou R, Horvath P, Siksnys V (2012). Cas9-crRNA ribonucleoprotein complex mediates specific DNA cleavage for adaptive immunity in bacteria. Proc Natl Acad Sci USA.

[31] Jacobs TB, LaFayette PR, Schmitz RJ, Parrott WA (2015). Targeted genome modifications in soybean with CRISPR/Cas9. BMC Biotechnol.

[32] Makarova KS, Haft DH, Barrangou R, Brouns SJJ, Charpentier E, Horvath P (2011). Evolution and classification of the CRISPR-Cas systems. Nat Rev Microbiol.

[33] Makarova KS, Wolf YI, Alkhnbashi OS, Costa F, Shah SA, Saunders SJ (2015). An updated evolutionary classification of CRISPR-Cas systems. Nat Rev Microbiol.

[34] Charpentier E, Richter H, van der Oost J, White MF (2015). Biogenesis pathways of RNA guides in archaeal and bacterial CRISPR-Cas adaptive immunity. FEMS Microbiol Rev.

[35] Shmakov S, Abudayyeh OO, Makarova KS, Wolf YI, Gootenberg JS, Semenova E (2015). Discovery and functional characterization of diverse class 2 CRISPR-Cas systems. Mol Cell.

[36] Makarova KS, Koonin EV (2015). Annotation and classification of CRISPR-Cas systems. Methods Mol Biol.

[37] Koonin EV, Makarova KS, Zhang F (2017). Diversity, classification and evolution of CRISPR-Cas systems. Curr Opin Microbiol.

[38] Lewis KM, Ke A (2017). Building the class 2 CRISPR-Cas arsenal. Mol Cell.

[39] Mali P, Yang L, Esvelt KM, Aach J, Guell M, DiCarlo JE (2013). RNA-guided human genome engineering via Cas9. Science.

[40] Deveau H, Barrangou R, Garneau JE, Labonté J, Fremaux C, Boyaval P (2008). Phage response to CRISPR-encoded resistance in *Streptococcus thermophilus*. J Bacteriol.

[41] Kleinstiver BP, Prew MS, Tsai SQ, Topkar VV, Nguyen NT, Zheng Z (2015). Engineered CRISPR-Cas9 nucleases with altered PAM specificities. Nature.

[42] Casini A, Olivieri M, Petris G, Montagna C, Reginato G, Maule G (2018). A highly specific SpCas9 variant is identified by *in vivo* screening in yeast. Nat Biotechnol.

[43] Walton RT, Christie KA, Whittaker MN, Kleinstiver BP (2020). Unconstrained genome targeting with near-PAMless engineered CRISPR-Cas9 variants. Science.

[44] Hsu PD, Scott DA, Weinstein JA, Ran FA, Konermann S, Agarwala V (2013). DNA targeting specificity of RNA-guided Cas9 nucleases. Nat Biotechnol.

[45] Ran FA, Cong L, Yan WX, Scott DA, Gootenberg JS, Kriz AJ (2015). In vivo genome editing using *Staphylococcus aureus* Cas9. Nature.

[46] Pausch P, Al-Shayeb B, Bisom-Rapp E, Tsuchida CA, Li Z, Cress BF (2020). CRISPR- CasΦ from huge phages is a hypercompact genome editor. Science.

[47] Chatterjee P, Jakimo N, Jacobson JM (2018). Minimal PAM specificity of a highly similar SpCas9 ortholog. Sci Adv.

[48] Hou Z, Zhang Y, Propson NE, Howden SE, Chu L-F, Sontheimer EJ (2013). Efficient genome engineering in human pluripotent stem cells using Cas9 from *Neisseria meningitidis*. Proc Natl Acad Sci USA.

[49] Kim E, Koo T, Park SW, Kim D, Kim K, Cho H-Y (2017). In vivo genome editing with a small Cas9 orthologue derived from *Campylobacter jejuni*. Nat Commun.

[50] Yamada M, Watanabe Y, Gootenberg JS, Hirano H, Ran FA, Nakane T (2017). Crystal structure of the minimal Cas9 from *Campylobacter jejuni* reveals the molecular diversity in the CRISPR-Cas9 systems. Mol Cell.

[51] Müller M, Lee CM, Gasiunas G, Davis TH, Cradick TJ, Siksnys V (2016). *Streptococcus thermophilus* CRISPR-Cas9 systems enable specific editing of the human genome. Mol Ther.

[52] Price AA, Sampson TR, Ratner HK, Grakoui A, Weiss DS (2015). Cas9-mediated targeting of viral RNA in eukaryotic cells. Proc Natl Acad Sci USA.

[53] Hirano H, Gootenberg JS, Horii T, Abudayyeh OO, Kimura M, Hsu PD (2016). Structure and engineering of *Francisella novicida* Cas9. Cell.

[54] Esvelt KM, Mali P, Braff JL, Moosburner M, Yaung SJ, Church GM (2013). Orthogonal Cas9 proteins for RNA-guided gene regulation and editing. Nat Methods.

[55] Chatterjee P, Lee J, Nip L, Koseki SRT, Tysinger E, Sontheimer EJ (2020). A Cas9 with PAM recognition for adenine dinucleotides. Nat Commun.

[56] Karvelis T, Gasiunas G, Young J, Bigelyte G, Silanskas A, Cigan M (2015). Rapid characterization of CRISPR-Cas9 protospacer adjacent motif sequence elements. Genome Biol.

[57] Burstein D, Harrington LB, Strutt SC, Probst AJ, Anantharaman K, Thomas BC (2017). New CRISPR-Cas systems from uncultivated microbes. Nature.

[58] Yang H, Patel DJ (2017). New CRISPR-Cas systems discovered. Cell Res.

[59] Hu JH, Miller SM, Geurts MH, Tang W, Chen L, Sun N (2018). Evolved Cas9 variants with broad PAM compatibility and high DNA specificity. Nature.

[60] Hu X, Wang C, Fu Y, Liu Q, Jiao X, Wang K (2016). Expanding the range of CRISPR/Cas9 genome editing in rice. Mol Plant.

[61] Nishimasu H, Ran FA, Hsu PD, Konermann S, Shehata SI, Dohmae N (2014). Crystal structure of Cas9 in complex with guide RNA and target DNA. Cell.

[62] Nishimasu H, Shi X, Ishiguro S, Gao L, Hirano S, Okazaki S (2018). Engineered CRISPR-Cas9 nuclease with expanded targeting space. Science.

[63] Xu Z, Kuang Y, Ren B, Yan D, Yan F, Spetz C (2021). SpRY greatly expands the genome editing scope in rice with highly flexible PAM recognition. Genome Biol.

[64] Kleinstiver BP, Pattanayak V, Prew MS, Tsai SQ, Nguyen NT, Zheng Z (2016). High-fidelity CRISPR-Cas9 nucleases with no detectable genome-wide off-target effects. Nature.

[65] Slaymaker IM, Gao L, Zetsche B, Scott DA, Yan WX, Zhang F (2016). Rationally engineered Cas9 nucleases with improved specificity. Science.

[66] Zeng D, Li X, Huang J, Li Y, Cai S, Yu W (2020). Engineered Cas9 variant tools expand targeting scope of genome and base editing in rice. Plant Biotechnol J.

[67] Chen JS, Dagdas YS, Kleinstiver BP, Welch MM, Sousa AA, Harrington LB (2017). Enhanced proofreading governs CRISPR-Cas9 targeting accuracy. Nature.

[68] Vakulskas CA, Dever DP, Rettig GR, Turk R, Jacobi AM, Collingwood MA (2018). A high-fidelity Cas9 mutant delivered as a ribonucleoprotein complex enables efficient gene editing in human hematopoietic stem and progenitor cells. Nat Med.

[69] Kleinstiver BP, Prew MS, Tsai SQ, Nguyen NT, Topkar VV, Zheng Z (2015). Broadening the targeting range of *Staphylococcus aureus* CRISPR-Cas9 by modifying PAM recognition. Nat Biotechnol.

[70] Tan Y, Chu AHY, Bao S, Hoang DA, Kebede FT, Xiong W (2019). Rationally engineered *Staphylococcus aureus* Cas9 nucleases with high genome-wide specificity. Proc Natl Acad Sci USA.

[71] Xie H, Ge X, Yang F, Wang B, Li S, Duan J (2020). High-fidelity SaCas9 identified by directional screening in human cells. PLoS Biol.

[72] Lee JK, Jeong E, Lee J, Jung M, Shin E, Kim Y-H (2018). Directed evolution of CRISPR-Cas9 to increase its specificity. Nat Commun.

[73] Chatterjee P, Jakimo N, Lee J, Amrani N, Rodríguez T, Koseki SRT (2020). An engineered ScCas9 with broad PAM range and high specificity and activity. Nat Biotechnol.

[74] Liu T, Zeng D, Zheng Z, Lin Z, Xue Y, Li T (2021). The ScCas9^++^ variant expands the CRISPR toolbox for genome editing in plants. J Integr Plant Biol.

[75] Ran FA, Hsu PD, Lin C-Y, Gootenberg JS, Konermann S, Trevino AE (2013). Double nicking by RNA-guided CRISPR Cas9 for enhanced genome editing specificity. Cell.

[76] Qi LS, Larson MH, Gilbert LA, Doudna JA, Weissman JS, Arkin AP (2021). Repurposing CRISPR as an RNA-guided platform for sequence-specific control of gene expression. Cell.

[77] Gilbert LA, Larson MH, Morsut L, Liu Z, Brar GA, Torres SE (2013). CRISPR-mediated modular RNA-guided regulation of transcription in eukaryotes. Cell.

[78] Gilbert LA, Horlbeck MA, Adamson B, Villalta JE, Chen Y, Whitehead EH (2014). Genome-scale CRISPR-mediated control of gene repression and activation. Cell.

[79] Dominguez AA, Lim WA, Qi LS (2016). Beyond editing: repurposing CRISPR-Cas9 for precision genome regulation and interrogation. Nat Rev Mol Cell Biol.

[80] Larson MH, Gilbert LA, Wang X, Lim WA, Weissman JS, Qi LS (2013). CRISPR interference (CRISPRi) for sequence-specific control of gene expression. Nat Protoc.

[81] Gupta D, Bhattacharjee O, Mandal D, Sen MK, Dey D, Dasgupta A (2019). CRISPR-Cas9 system: a new-fangled dawn in gene editing. Life Sci..

[82] Jensen NB, Strucko T, Kildegaard KR, David F, Maury J, Mortensen UH (2014). EasyClone: method for iterative chromosomal integration of multiple genes in *Saccharomyces cerevisiae*. FEMS Yeast Res.

[83] Kungulovski G, Jeltsch A (2016). Epigenome editing: state of the art, concepts, and perspectives. Trends Genet.

[84] Chen B, Gilbert LA, Cimini BA, Schnitzbauer J, Zhang W, Li G-W (2013). Dynamic imaging of genomic loci in living human cells by an optimized CRISPR/Cas system. Cell.

[85] Barman A, Deb B, Chakraborty S (2020). A glance at genome editing with CRISPR-Cas9 technology. Curr Genet.

[86] Komor AC, Kim YB, Packer MS, Zuris JA, Liu DR (2016). Programmable editing of a target base in genomic DNA without double-stranded DNA cleavage. Nature.

[87] Nishida K, Arazoe T, Yachie N, Banno S, Kakimoto M, Tabata M (2016). Targeted nucleotide editing using hybrid prokaryotic and vertebrate adaptive immune systems. Science.

[88] Ma Y, Zhang J, Yin W, Zhang Z, Song Y, Chang X (2016). Targeted AID-mediated mutagenesis (TAM) enables efficient genomic diversification in mammalian cells. Nat Methods.

[89] Gaudelli NM, Komor AC, Rees HA, Packer MS, Badran AH, Bryson DI (2017). Programmable base editing of A•T to G•C in genomic DNA without DNA cleavage. Nature.

[90] Grünewald J, Zhou R, Lareau CA, Garcia SP, Iyer S, Miller BR (2020). A dual-deaminase CRISPR base editor enables concurrent adenine and cytosine editing. Nat Biotechnol.

[91] Sakata RC, Ishiguro S, Mori H, Tanaka M, Tatsuno K, Ueda H (2020). Base editors for simultaneous introduction of C-to-T and A-to-G mutations. Nat Biotechnol.

[92] Xie J, Huang X, Wang X, Gou S, Liang Y, Chen F (2020). ACBE, a new base editor for simultaneous C-to-T and A-to-G substitutions in mammalian systems. BMC Biol.

[93] Zhang X, Zhu B, Chen L, Xie L, Yu W, Wang Y (2020). Dual base editor catalyzes both cytosine and adenine base conversions in human cells. Nat Biotechnol.

[94] Kurt IC, Zhou R, Iyer S, Garcia SP, Miller BR, Langner LM (2021). CRISPR C-to-G base editors for inducing targeted DNA transversions in human cells. Nat Biotechnol.

[95] Zhao D, Li J, Li S, Xin X, Hu M, Price MA (2021). Glycosylase base editors enable C-to-A and C-to-G base changes. Nat Biotechnol.

[96] Zeng D, Liu T, Tan J, Zhang Y, Zheng Z, Wang B (2020). PhieCBEs: plant high-efficiency cytidine base editors with expanded target range. Mol Plant.

[97] Anzalone AV, Randolph PB, Davis JR, Sousa AA, Koblan LW, Levy JM (2019). Search-and-replace genome editing without double-strand breaks or donor DNA. Nature.

[98] Zetsche B, Gootenberg JS, Abudayyeh OO, Slaymaker IM, Makarova KS, Essletzbichler P (2015). Cpf1 is a single RNA-guided endonuclease of a class 2 CRISPR-Cas system. Cell.

[99] Chylinski K, Le Rhun A, Charpentier E (2013). The tracrRNA and Cas9 families of type II CRISPR-Cas immunity systems. RNA Biol.

[100] Tu M, Lin L, Cheng Y, He X, Sun H, Xie H (2017). A “new lease of life”: FnCpf1 possesses DNA cleavage activity for genome editing in human cells. Nucleic Acids Res.

[101] Li Z, Xiong X, Li J-F (2018). New cytosine base editor for plant genome editing. Sci China Life Sci.

[102] Alok A, Sandhya D, Jogam P, Rodrigues V, Bhati KK, Sharma H (2020). The rise of the CRISPR/Cpf1 system for efficient genome editing in plants. Front Plant Sci.

[103] Endo A, Masafumi M, Kaya H, Toki S (2016). Efficient targeted mutagenesis of rice and tobacco genomes using Cpf1 from *Francisella novicida*. Sci Rep.

[104] Gao L, Cox DBT, Yan WX, Manteiga JC, Schneider MW, Yamano T (2017). Engineered Cpf1 variants with altered PAM specificities. Nat Biotechnol.

[105] Li S, Zhang X, Wang W, Guo X, Wu Z, Du W (2018). Expanding the scope of CRISPR/Cpf1-mediated genome editing in rice. Mol Plant.

[106] Zhong Z, Zhang Y, You Q, Tang X, Ren Q, Liu S (2018). Plant genome editing using FnCpf1 and LbCpf1 nucleases at redefined and altered PAM sites. Mol Plant.

[107] Ming M, Ren Q, Pan C, He Y, Zhang Y, Liu S (2020). CRISPR-Cas12b enables efficient plant genome engineering. Nat Plants.

[108] Wang Q, Alariqi M, Wang F, Li B, Ding X, Rui H (2020). The application of a heat-inducible CRISPR/Cas12b (C2c1) genome editing system in tetraploid cotton (*G. hirsutum*) plants. Plant Biotechnol J.

[109] Shmakov S, Smargon A, Scott D, Cox D, Pyzocha N, Yan W (2017). Diversity and evolution of class 2 CRISPR-Cas systems. Nat Rev Microbiol.

[110] Abudayyeh OO, Gootenberg JS, Essletzbichler P, Han S, Joung J, Belanto JJ (2017). RNA targeting with CRISPR-Cas13. Nature.

[111] Cox DBT, Gootenberg JS, Abudayyeh OO, Franklin B, Kellner MJ, Joung J (2017). RNA editing with CRISPR-Cas13. Science.

[112] Abudayyeh OO, Gootenberg JS, Konermann S, Joung J, Slaymaker IM, Cox DBT (2016). C2c2 is a single-component programmable RNA-guided RNA-targeting CRISPR effector. Science.

[113] East-Seletsky A, O’Connell MR, Knight SC, Burstein D, Cate JHD, Tjian R (2016). Two distinct RNase activities of CRISPR-C2c2 enable guide-RNA processing and RNA detection. Nature.

[114] Abudayyeh OO, Gootenberg JS, Franklin B, Koob J, Kellner MJ, Ladha A (2019). A cytosine deaminase for programmable single-base RNA editing. Science.

[115] Kannan S, Altae-Tran H, Jin X, Madigan VJ, Oshiro R, Makarova KS (2021). Compact RNA editors with small Cas13 proteins. Nat Biotechnol.

[116] Aman R, Ali Z, Butt H, Mahas A, Aljedaani F, Khan MZ (2018). RNA virus interference via CRISPR/Cas13a system in plants. Genome Biol.

[117] Ren J, Fu L, Nile SH, Zhang J, Kai G (2019). *Salvia miltiorrhiza* in treating cardiovascular diseases: a review on its pharmacological and clinical applications. Front Pharmacol.

[118] Luo H, Zhu Y, Song J, Xu L, Sun C, Zhang X (2014). Transcriptional data mining of *Salvia miltiorrhiza* in response to methyl jasmonate to examine the mechanism of bioactive compound biosynthesis and regulation. Physiol Plant.

[119] Li B, Cui G, Shen G, Zhan Z, Huang L, Chen J (2017). Targeted mutagenesis in the medicinal plant *Salvia miltiorrhiza*. Sci Rep.

[120] Zhou Z, Tan H, Li Q, Chen J, Gao S, Wang Y (2018). CRISPR/Cas9-mediated efficient targeted mutagenesis of RAS in *Salvia miltiorrhiza*. Phytochemistry.

[121] Zhou Z, Li Q, Xiao L, Wang Y, Feng J, Bu Q (2021). Multiplexed CRISPR/Cas9-mediated knockout of laccase genes in *Salvia miltiorrhiza* revealed their roles in growth, development, and metabolism. Front Plant Sci.

[122] Shi M, Du Z, Hua Q, Kai G (2021). CRISPR/Cas9-mediated targeted mutagenesis of *bZIP2* in *Salvia miltiorrhiza* leads to promoted phenolic acid biosynthesis. Ind Crops Prod.

[123] Liang J, Chen S, Hu Y, Yang Y, Yuan J, Wu Y (2018). Protective roles and mechanisms of *Dendrobium officinal* polysaccharides on secondary liver injury in acute colitis. Int JBiol Macromol.

[124] Liang J, Li H, Chen J, He L, Du X, Zhou L (2019). *Dendrobium officinale* polysaccharides alleviate colon tumorigenesis via restoring intestinal barrier function and enhancing anti-tumor immune response. Pharmacol Res.

[125] Chen H, Yin J, Yeh C, Lu Y, Yang J (2020). Inverse synthetic aperture radar imaging based on time–frequency analysis through neural network. J Electron Imaging.

[126] Zhang L-J, Huang X-J, Shi X-D, Chen H-H, Cui SW, Nie S-P (2019). Protective effect of three glucomannans from different plants against DSS induced colitis in female BALB/c mice. Food Funct.

[127] Yang K, Lu T, Zhan L, Zhou C, Zhang N, Lei S (2020). Physicochemical characterization of polysaccharide from the leaf of *Dendrobium officinale* and effect on LPS induced damage in GES-1 cell. Int J Biol Macromol.

[128] Hou B, Tian M, Luo J, Ji Y, Xue Q, Ding X (2012). Genetic diversity assessment and ex situ conservation strategy of the endangered *Dendrobium officinale* (Orchidaceae) using new trinucleotide microsatellite markers. Plant Syst Evol.

[129] Ren R, Gao J, Yin D, Li K, Lu C, Ahmad S (2021). Highly efficient Leaf base protoplast isolation and transient expression systems for orchids and other important monocot crops. Front Plant Sci.

[130] da Silva JAT, Dobránszki J, Cardoso JC, Chandler SF, Zeng S (2016). Methods for genetic transformation in *Dendrobium*. Plant Cell Rep.

[131] Kui L, Chen H, Zhang W, He S, Xiong Z, Zhang Y (2016). Building a genetic manipulation tool box for orchid biology: identification of constitutive promoters and application of CRISPR/Cas9 in the orchid *Dendrobium officinale*. Front Plant Sci.

[132] Li Y, Zhang B, Yu H (2021). Kilobase-scale genomic deletion of *DOTFL1* in *dendrobium* orchids. J Genet Genomics.

[133] Devsi A, Kiyota B, Ouellette T, Hegle AP, Rivera-Acevedo RE, Wong J (2020). A pharmacological characterization of *Cannabis sativa* chemovar extracts. J Cannabis Res.

[134] Schultz CJ, Lim WL, Khor SF, Neumann KA, Schulz JM, Ansari O (2020). Consumer and health-related traits of seed from selected commercial and breeding lines of industrial hemp, *Cannabis sativa* L. J Agr Food Res..

[135] Malinowska B, Baranowska-Kuczko M, Kicman A, Schlicker E (2021). Opportunities, challenges and pitfalls of using cannabidiol as an adjuvant drug in COVID-19. Int J Mol Sci.

[136] Aliekperova N, Kosyachenko К, Kaniura O (2020). Perspectives on formation of medical cannabis market in Ukraine based on holistic approach. J Cannabis Res.

[137] Sorokin A, Yadav NS, Gaudet D, Kovalchuk I (2020). Transient expression of the *β-glucuronidase* gene in *Cannabis sativa* varieties. Plant Signal Behav.

[138] Ahmed S, Gao X, Jahan MA, Adams M, Wu N, Kovinich N (2021). Nanoparticle-based genetic transformation of *Cannabis sativa*. J Biotechnol.

[139] Zhang X, Xu G, Cheng C, Lei L, Sun J, Xu Y (2021). Establishment of an *Agrobacterium*-mediated genetic transformation and CRISPR/Cas9-mediated targeted mutagenesis in Hemp (*Cannabis Sativa* L.). Plant Biotechnol J.

[140] Staiger C (2012). Comfrey: a clinical overview. Phytother Res.

[141] Stickel F, Seitz HK (2000). The efficacy and safety of comfrey. Public Health Nutr.

[142] Allgaier C, Franz S (2015). Risk assessment on the use of herbal medicinal products containing pyrrolizidine alkaloids. Regul Toxicol Pharmacol.

[143] Knutsen HK, Alexander J, Barregård L, Bignami M, Brüschweiler B, EFSA Panel on Contaminants in the Food Chain (CONTAM) (2017). Risks for human health related to the presence of pyrrolizidine alkaloids in honey, tea, herbal infusions and food supplements. EFSA J.

[144] Kopp T, Abdel-Tawab M, Mizaikoff B (2020). Extracting and analyzing pyrrolizidine alkaloids in medicinal plants: a review. Toxins.

[145] Zakaria MM, Schemmerling B, Ober D (2021). CRISPR/Cas9-mediated genome editing in comfrey (*Symphytum officinale*) hairy roots results in the complete eradication of pyrrolizidine alkaloids. Molecules.

[146] Alagoz Y, Gurkok T, Zhang B, Unver T (2016). Manipulating the biosynthesis of bioactive compound alkaloids for next-generation metabolic engineering in opium poppy using CRISPR-Cas 9 genome editing technology. Sci Rep.

[147] Feng S, Song W, Fu R, Zhang H, Xu A, Li J (2018). Application of the CRISPR/Cas9 system in *Dioscorea zingiberensis*. Plant Cell Tiss Organ Cult.

[148] Ro D-K, Paradise EM, Ouellet M, Fisher KJ, Newman KL, Ndungu JM (2006). Production of the antimalarial drug precursor artemisinic acid in engineered yeast. Nature.

[149] Malhotra K, Subramaniyan M, Rawat K, Kalamuddin M, Qureshi MI, Malhotra P (2016). Compartmentalized metabolic engineering for artemisinin biosynthesis and effective malaria treatment by oral delivery of plant cells. Mol Plant.

[150] Li J, Mutanda I, Wang K, Yang L, Wang J, Wang Y (2019). Chloroplastic metabolic engineering coupled with isoprenoid pool enhancement for committed taxanes biosynthesis in *Nicotiana benthamiana*. Nat Commun.

[151] Zhou K, Qiao K, Edgar S, Stephanopoulos G (2015). Distributing a metabolic pathway among a microbial consortium enhances production of natural products. Nat Biotechnol.

[152] Hu T, Zhou J, Tong Y, Su P, Li X, Liu Y (2020). Engineering chimeric diterpene synthases and isoprenoid biosynthetic pathways enables high-level production of miltiradiene in yeast. Metab Eng.

[153] Zhou YJ, Gao W, Rong Q, Jin G, Chu H, Liu W (2012). Modular pathway engineering of diterpenoid synthases and the mevalonic acid pathway for miltiradiene production. J Am Chem Soc.

[154] Luo X, Reiter MA, d’Espaux L, Wong J, Denby CM, Lechner A (2019). Complete biosynthesis of cannabinoids and their unnatural analogues in yeast. Nature.

[155] Zhang D, Hussain A, Manghwar H, Xie K, Xie S, Zhao S (2020). Genome editing with the CRISPR-Cas system: an art, ethics and global regulatory perspective. Plant Biotechnol J.

[156] Waltz E (2018). With a free pass, CRISPR-edited plants reach market in record time. Nat Biotechnol.

[157] Gupta M, Gerard M, Padmaja SS, Sastry RK (2020). Trends of CRISPR technology development and deployment into agricultural production-consumption systems. World Pat Inf.

[158] Zhou Q, Liu W, Zhang Y, Liu KK (2007). Action mechanisms of acetolactate synthase-inhibiting herbicides. Pestic Biochem Physiol.

[159] Kaundun SS (2014). Resistance to acetyl-CoA carboxylase-inhibiting herbicides. Pest Manag Sci.

[160] Hummel AW, Chauhan RD, Cermak T, Mutka AM, Vijayaraghavan A, Boyher A (2018). Allele exchange at the EPSPS locus confers glyphosate tolerance in cassava. Plant Biotechnol J.

[161] de Pater S, Klemann BJPM, Hooykaas PJJ (2018). True gene-targeting events by CRISPR/Cas-induced DSB repair of the PPO locus with an ectopically integrated repair template. Sci Rep.

[162] Liu L, Kuang Y, Yan F, Li S, Ren B, Gosavi G (2021). Developing a novel artificial rice germplasm for dinitroaniline herbicide resistance by base editing of *OsTubA2*. Plant Biotechnol J.

[163] Butt H, Eid A, Momin AA, Bazin J, Crespi M, Arold ST (2019). CRISPR directed evolution of the spliceosome for resistance to splicing inhibitors. Genome Biol.

[164] Religia P, Nguyen ND, Nong QD, Matsuura T, Kato Y, Watanabe H (2021). Mutation of the cytochrome P450 *CYP360A8* gene increases sensitivity to paraquat in *Daphnia magna*. Environ Toxicol Chem.

[165] Li T, Yang X, Yu Y, Si X, Zhai X, Zhang H (2018). Domestication of wild tomato is accelerated by genome editing. Nat Biotechnol.

[166] Ma Y, Cui G, Chen T, Ma X, Wang R, Jin B (2021). Expansion within the CYP71D subfamily drives the heterocyclization of tanshinones synthesis in *Salvia miltiorrhiza*. Nat Commun.

[167] Zhang J, Lv H, Liu W, Ji A, Zhang X, Song J (2020). bHLH transcription factor *SmbHLH92* negatively regulates biosynthesis of phenolic acids and tanshinones in *Salvia miltiorrhiza*. Chin Herb Med.

[168] Zhang Y, Ji A, Xu Z, Luo H, Song J (2019). The AP2/ERF transcription factor SmERF128 positively regulates diterpenoid biosynthesis in *Salvia miltiorrhiza*. Plant Mol Biol.

[169] Yu H, Guo W, Yang D, Hou Z, Liang Z (2018). Transcriptional profiles of *SmWRKY* family genes and their putative roles in the biosynthesis of tanshinone and phenolic acids in *Salvia miltiorrhiza*. Int J Mol Sci.

[170] Demirer GS, Zhang H, Matos JL, Goh NS, Cunningham FJ, Sung Y (2019). High aspect ratio nanomaterials enable delivery of functional genetic material without DNA integration in mature plants. Nat Nanotechnol.

[171] Kwak S-Y, Lew TTS, Sweeney CJ, Koman VB, Wong MH, Bohmert-Tatarev K (2019). Chloroplast-selective gene delivery and expression in planta using chitosan-complexed single-walled carbon nanotube carriers. Nat Nanotechnol.

[172] Zhang H, Demirer GS, Zhang H, Ye T, Goh NS, Aditham AJ (2019). DNA nanostructures coordinate gene silencing in mature plants. Proc Natl Acad Sci USA.

[173] Santana I, Wu H, Hu P, Giraldo JP (2020). Targeted delivery of nanomaterials with chemical cargoes in plants enabled by a biorecognition motif. Nat Commun.

[174] Ma X, Zhang X, Liu H, Li Z (2020). Highly efficient DNA-free plant genome editing using virally delivered CRISPR-Cas9. Nat Plants.

